# Simulation-based biomechanical assessment of unpowered exoskeletons for running

**DOI:** 10.1038/s41598-021-89640-3

**Published:** 2021-06-04

**Authors:** Hamidreza Aftabi, Rezvan Nasiri, Majid Nili Ahmadabadi

**Affiliations:** Cognitive Systems Laboratory, Control and Intelligent Processing Center of Excellence (CIPCE), School of Electrical and Computer Engineering, College of Engineering, University of Tehran, Tehran, Iran

**Keywords:** Biomedical engineering, Electromyography - EMG, Muscle, Musculoskeletal system, Motor control, Neuromuscular junction, Metabolism

## Abstract

Due to the complexity and high degrees of freedom, the detailed assessment of human biomechanics is necessary for the design and optimization of an effective exoskeleton. In this paper, we present full kinematics, dynamics, and biomechanics assessment of unpowered exoskeleton augmentation for human running gait. To do so, the considered case study is the assistive torque profile of *I-RUN*. Our approach is using some extensive data-driven OpenSim simulation results employing a generic lower limb model with 92-muscles and 29-DOF. In the simulation, it is observed that exoskeleton augmentation leads to $4.62\%$ metabolic rate reduction for the stiffness coefficient of $\alpha ^*=0.6$. Moreover, this optimum stiffness coefficient minimizes the biological hip moment by $26\%$. The optimum stiffness coefficient ($\alpha ^*=0.6$) also reduces the average force of four major hip muscles, i.e., Psoas, Gluteus Maximus, Rectus Femoris, and Semimembranosus. The effect of assistive torque profile on the muscles’ fatigue is also studied. Interestingly, it is observed that at $\alpha ^{\#}=0.8$, both all 92 lower limb muscles’ fatigue and two hip major mono-articular muscles’ fatigue have the maximum reduction. This result re-confirm our hypothesis that ”reducing the forces of two antagonistic mono-articular muscles is sufficient for involved muscles’ total fatigue reduction.” Finally, the relation between the amount of metabolic rate reduction and kinematics of hip joint is examined carefully where for the first time, we present a reliable kinematic index for prediction of the metabolic rate reduction by *I-RUN* augmentation. This index not only explains individual differences in metabolic rate reduction but also provides a quantitative measure for training the subjects to maximize their benefits from *I-RUN*.

## Introduction

Passive exoskeletons for metabolic rate reduction in running and walking are popular in the research community because of their innovative design as well as the perspective of usability, low weight, and affordable cost^1,4–9^. Nevertheless, as for the active ones, passive exoskeletons have not realized the considered expectations in terms of efficiency, rehabilitation, and so on. The main reason is the lack of a comprehensive model for accurate user-exoskeleton interaction; e.g., predictive forward dynamic models^10–12^. That chiefly stems from high redundancy in our musculoskeletal system^13–15^ and significant individual differences^16,17^, as well as the complexity of our neural control system^18,19^. As a result, there is no specific and straight-forward method for an exoskeleton design, estimation of its efficacy for a potential user, and on-line adaptation to individuals. Therefore, extensive experiments, laboratory measurements, hand-tunings, and user training are required to benefit from an exoskeleton.

The existing unpowered assistive devices are equipped with elastic elements to save energy in a certain phase of motion and recycle it during another phase. The assistive torque applied by an elastic element is a function of joints’ angles. Therefore, the performance of these devices in metabolic rate reduction and comfort increment is very sensitive to users’ kinematic and the compliance profiles of elastic parts. As a result, (1) adaptation of compliant elements (devising a semi-passive exoskeleton)^20,21^ as well as (2) modification of subjects’ kinematic by training^4^ are two solutions for improving the performance of a full-passive exoskeleton. To materialize these two approaches for an assistive device, a low-cost and simple compliance adaptation method and a quantitative straight-forward kinematic measure for subject training are required. Besides, due to the complexity of measuring metabolic rate, evaluating the relationship between metabolic rate and biomechanical parameters has gained much attention^22–24^. Such a kinematic measure can also be potentially employed to estimate exoskeletons’ metabolic rate reduction for an individual as well; it is a substitute for laboratory-bounded costly devices like gas analyzers. In this paper, we target these requirements for our previously designed exoskeleton device named *I-RUN*^1^.

Previously, we reported the first unpowered exoskeleton for metabolic rate reduction during running; on average $8\pm 1.5 \%$ at 2.5*m*/*s*^1^. However, we observed high individual differences in metabolic rate reduction, and there was no clear explanation for that. The only thing we knew was that there is a certain relation between the metabolic rate reduction, the compliance coefficient, and the subjects’ kinematic during running. To investigate and exploit that relation for the above-mentioned purposes, we employ some extensive detailed simulations.

Using simulations for exoskeleton design and analysis as a partial substitute for costly experiments has gained momentum in recent years^25–29^. For instance, based on simulation results, it is suggested that a shifted exoskeleton torque profile for the hip joint is very effective for reducing the metabolic cost of running^27^; based on that, an active exosuit device was devised^30^. As another example, Jackson *et al.*^29^ explained the exoskeleton effects on the biomechanical parameters by a forward simulation approach.

Here, the same strategy is taken, and by using a simulation-based approach, we investigate the relationship between metabolic rate reduction and other contributors; namely elastic element stiffness, exoskeleton torque profile, biological moment, muscles’ forces, and gait kinematic. Accordingly, a simple kinematic index for subject training and estimation of metabolic rate reduction is devised. This index explains individual differences in metabolic rate reduction and provides a quantitative measure for subjects to maximize their benefits from *I-RUN*. Besides, we study if there is a relation between the metabolic rate and all muscles’ fatigue. Computation of all muscles’ fatigue requires each muscle’s electromyography (EMG) signal, which makes it inapplicable in practice. To resolve this problem, inspired by Nasiri *et al.*^31^, we test our hypothesis that ”the fatigue of two antagonistic mono-articular muscles at the target joint has a linear correlation with all muscles’ fatigue.” Having this hypothesis validated, it is shown that reducing the EMG signal of only two antagonistic mono-articular muscles is sufficient for whole muscles’ fatigue reduction. In addition, it is demonstrated that the compliance value resulting in the minimum effort of two antagonistic mono-articular muscles is a good sub-optimal for the metabolic rate reduction. These findings simplify online exoskeleton adaptation.

## Methods and materials

### Experimental data

Dataset of 10 subjects reported by Hamner *et al.*^32^, was used in OpenSim^3^ to run a muscle driven simulation. Due to technical issues, we were only able to use the data of 7 subjects in OpenSim 4.0. As mentioned by Hamner *et al.*^32^, subjects have an average age, height, and mass of $29\pm {5}y$, $177\pm {4}cm$ , and $70.9\pm {7.0}kg$, respectively. This dataset contains marker positions and ground reaction forces (GRF) of running gait at four different speeds: 2.0, 3.0, 4.0, and 5.0*m*/*s*. It is important to note that the aim of this paper is to study the biomechanical effects of *I-RUN* augmentation regardless of the user running speed. It is also worth mentioning that *I-RUN* is designed for a range of running speed, and it is not restricted to a specific running speed; in Nasiri *et al.*^1^, the experimental validation was at 2.5*m*/*s*. Nevertheless, to have a comparative connection between the experimental results of *I-RUN* and this simulation study, we had to choose either 2.0*m*/*s* or 3.0*m*/*s*. As it is reported by Schache *et al.*,^33^, for speeds higher than 2.5*m*/*s*, the posture, dynamics, and trajectory of the joints are significantly changed; hence, 2*m*/*s* was chosen for our simulation study.

### Dynamic simulations

We used a 12 segment musculoskeletal model with 29 DOF^34^ in OpenSim to run a musculoskeletal simulation; this model covers frontal, lateral, and sagittal movements. The lower extremity and back joints of this model were actuated by 92 hill-type muscle-tendon units^35,36^, and arms were controlled by ideal actuators. The generic model was scaled to create a model consistent with each subject’s anthropometry. Scaling was done by minimizing the difference between experimental markers (the experimental markers are placed on anatomical landmarks of subjects) and their corresponding virtual markers (the virtual markers are placed on the generic model) in a static pose. To calculate each joint angle, we used an inverse kinematics procedure. Unlike scaling, which uses the marker trajectories of the static pose, the inverse kinematics algorithm uses the marker trajectories of the running gait as an input to minimize the difference between experimental and virtual markers.

**Figure 1:**
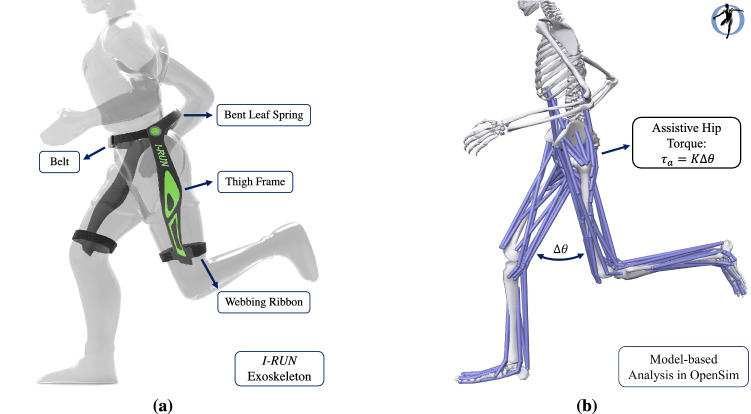
**Description of** ***I-RUN***. (**a**) describes the functionality of *I-RUN*^1,2^. Using *I-RUN* resulted in on average $8\%$ metabolic rate reduction in ten healthy active subjects at $2.5\,\mathrm{m/s}$ running speed. (**b**) shows our model-based approach for evaluating the efficacy of the *I-RUN* torque profile in OpenSim software^3^. Since the *I-RUN* torque is a linear function of the difference between hip angles, we have modeled it as $\tau_{a}= K \Delta \theta$, where $\tau_{a}$ represents the assistive torque, $\Delta \theta$ is the difference between left and right hip angles, and *K* is the stiffness.

Based on our experimental studies (torsion test on the I-Run bent-leaf-spring for deflections between $0\,deg$ up to $60\,deg$), for deflections lower than $30\,deg$, *I-RUN* applies torque to hip joints in linear relation to the difference between left and right hip angles. Accordingly, this mechanism couples two hips with each other using a torsional spring; i.e., Bent Leaf Spring (BLS) in Fig. 1a. To model this assistive device, two ideal coordinate actuators were added to the hip joints of our musculoskeletal model (Fig. 1b) . For evaluating our exoskeleton in different stiffnesses of BLS, we defined a factor named spring coefficient ($\alpha$); $\alpha$ scales the inverse dynamic stiffness ($K_{inv}$). $K_{inv}$ is the stiffness value for the exoskeleton device that minimizes $J=\tau _h^2$ where $\tau _h$ is the biological hip moment. In this case, the assistive torque of the exoskeleton is written as $\tau _a=\alpha K_{inv} \Delta \theta$ where $\tau _a$ represents the assistive torque, and $\Delta \theta$ is the difference between left and right hip angles. It is obvious that $\alpha =0$ and $\alpha =1$ refer to no assistive torque and full possible average hip moment compensation conditions, respectively.

We used the residual reduction algorithm (RRA)^3^ to calculate the moment of each joint. RRA algorithm modifies the torso mass center of the subject’s specific model. Besides, it allows for minor changes in joint angles (less than 2*deg*), previously calculated from the inverse kinematics, to create a model that is more compatible with the ground reaction forces dataset. We employed the computed muscle control algorithm (CMC)^37^ to calculate the muscles’ forces. CMC minimizes the sum of the square of muscles’ forces while driving the musculoskeletal model toward desired kinematics. CMC objective function can be written as^38^:

1 $$\begin{aligned} J\left( f,r,R\right)&=\sum _{i=1}^{n_M}\left( \frac{{f_i}}{\omega _i}\right) ^2+\sum _{j=1}^{n_r}\left( \frac{r_j}{\omega _j}\right) ^2+\sum _{k=1}^{n_R}\left( \frac{R_k}{\omega _k}\right) ^2 \end{aligned}$$

where *f*, *r*, and *R* are muscle forces, reserve torques, and residual torques, respectively; $n_M=92$, $n_r=29$, and $n_R=6$ . Besides, $\omega _{i,j,k}$ refer to their corresponding weights. Reserve actuators (*r*) are responsible for the model’s strength by applying small torques in joints, while residual actuators model 6 degrees of freedom (3 translational, 3 rotational) between the pelvis of the generic model and the ground. Weights of residual and reserve actuators are chosen small enough to ensure a high cost in the objective function.

### Data analysis

For each subject, the metabolic cost at each spring coefficient was calculated using the method presented by Uchida *et al.*^39^. The total metabolic cost was computed by integrating the instantaneous metabolic cost over one gait cycle and divided by the stride period. Besides, to normalize the results, the total metabolic cost was divided by each subject’s weight.

Average muscle force and moment were calculated by integrating the force and absolute moment of the time series data and divided by the stride period and the subject’s mass.

In this paper, normalized muscle force is defined as the muscle force divided by its maximum isometric force. In addition, the instantaneous all muscles’ fatigue is defined as the summation of the square of all normalized muscles’ forces^40,41^; please note that muscle fatigue may also be mentioned as muscles’ effort in the literature. And, the instantaneous antagonistic mono-articular muscles’ fatigue at the hip joint is the summation of the square of the normalized Psoas and Gluteus Maximus muscles’ forces. Finally, muscles’ fatigue was computed by integrating the instantaneous muscles’ fatigue over one gait cycle and divided by the stride period.

### Statistics

At each spring coefficient, means and s.e.ms of net metabolic rate, average joint moment, average muscle force and, muscle fatigue were calculated across the subjects. Due to previous observations that the metabolic rate is a nonlinear function of different stiffnesses^5,42^ and the hypothesis that the muscles’ fatigue is a quadratic function of muscles’ forces^40,41^, we performed a mixed model, three-factor ANOVA (random effect: participant; fixed effects: spring coefficient and the square of spring coefficient; significance level $=0.05$; JMP Pro) to evaluate the effect of the spring coefficients on the metabolic rate and muscles’ fatigue. We also carried out a two-factor ANOVA test (random effect: participant; fixed effects: spring coefficient; significance level $=0.05$; JMP Pro) to assess the effect of the spring coefficients on the joint moment and muscle force. Besides, a least-square method was employed to (1) fit a second-order polynomial to the mean data of the metabolic rate and muscles’ fatigue and (2) find the best linear relation between potential kinematic indexes and the metabolic rate reduction in the optimum stiffness.

For post hoc analysis, we utilized a paired two-sided *t-*test for multiple comparisons with Holm–Bonferroni correction (significance level $=0.05$; Scikit–Learn, Python 3.6) in order to compare the spring coefficients with each other and find out which spring coefficient yields a significant change in the net metabolic rate and muscle fatigue. Finally, a Shapiro–Wilk test (significance level $=0.05$; JMP Pro) was employed to ensure the appropriateness of the tests in which normality is a necessary condition.

### Results evaluation

Finally, to assess the validity of our results, we evaluated different parameters, such as peak residual moments, peak residual forces, and peak reserve actuator torques. In all conditions and for all subjects, maximum residual moments(forces) were less than 40*Nm*(12.5*N*); the maximum residual force was much less than $5\%$ of the peak magnitude of experimental GRF^43^. And, the peak of reserve actuator torques was less than 20*Nm*. Besides, by comparing the kinematics of simulations and experimental data, it is observed that the maximum RMS deviation is lower than 2*deg*. As it is mentioned before, the maker locations on the model were scaled to fit with the real dataset; RMS markers error did not exceed 2*cm*. All of these values were less than OpenSim best practice thresholds^44^, which approves the preciseness of our simulations.

### Assumptions, limitations, and considerations

**Weight:** The goal of this study is to assess the biomechanical effects of *I-RUN* torque profile augmentation. Hence, the *I-RUN* is modeled as ideal actuators applying torque as a linear function of angle. **Kinematic:** CMC algorithm cannot determine the possible kinematic changes by assistive torque augmentation. Accordingly, in the whole of this simulation study, it is assumed that the user kinematic is almost fixed with and without *I-RUN* torque augmentation. In some cases, exoskeleton augmentation has a considerable impact on the joints’ kinematic^24,45^; however, *I-RUN* is designed based on the dynamics of human running, and it does not perturb the natural dynamics of running, the joint’s total torque, and consequently the kinematics of running. Hence, in the *I-RUN* case, this restriction is a meaningful assumption. **Solver:** The main weakness of static optimization approaches is that they assume that the subject’s motions are almost fixed before and after adding/wearing an exoskeleton device. Hence, the results of this method are reliable for the cases that the joint trajectories are not significantly changed by exoskeleton augmentation. Besides, the static optimization methods suffer from the lack of smoothness in their extracted torques/forces profiles. Accordingly, the variation among different types of static optimization approaches comes from their data-driven solutions for resolving the biomechanical variables and their chosen cost function or redundancy resolution method. Among all of the static optimization toolboxes for neuromuscular model resolution, computed muscle control (CMC)^37^ provides smoother torques/forces profiles. And, due to the promising and reliable results of CMC, it has gained much attention in recent years for analyzing the biomechanical effects of adding exoskeleton devices. **Other limitations**: for the sake of simplicity and without losing generality, we did not model muscle fatigue and co-activation, assuming that the co-activations don’t change with and without *I-RUN* because of keeping the gate the same.

## Results and discussion

### Metabolic rate reduction

**Figure 2:**
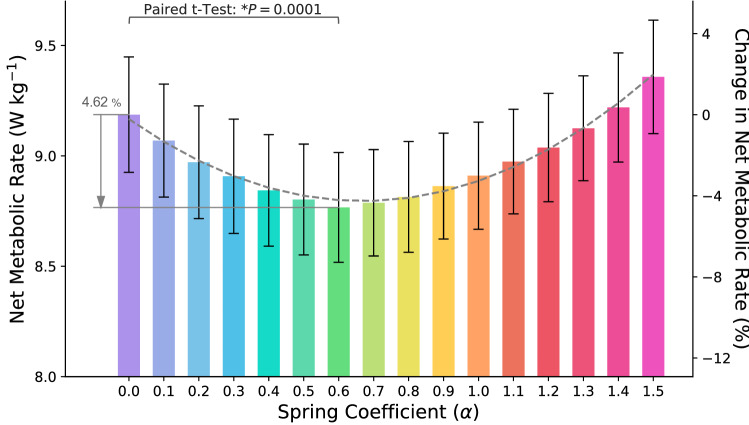
**Metabolic rate**. Metabolic rate versus the stiffness coefficient (N $=7$; ANOVA with second order model; random effect: participant; fixed effects: spring coefficient and square of spring coefficient; $P_{scoef}<0.0001$, $P_{scoef^2}<0.0001$). The maximum metabolic rate reduction is $4.62\pm 0.29\%$ (mean ± s.e.m) at $\alpha ^*=0.6$ (paired two-sided *t-*test with correction for multiple comparisons; $P=0.0001$). The dashed line is the quadratic best fitted profile to average metabolic costs ($R^2=0.98$, $P<0.0001$).

To find a metric for the metabolic rate reduction, as the first step, we compute the optimum stiffness for the metabolic rate reduction in our model. Accordingly, we plot the average metabolic cost of seven subjects (in Fig. 2) versus different spring coefficients; from $\alpha =0$ to $\alpha =1.5$ with the step size of 0.1. As can be seen in Fig. 2, the spring coefficient value that minimizes the metabolic rate is at $\alpha ^*=0.6$; the mean metabolic rate reduction at $\alpha ^*=0.6$ is $4.62\%$. It is worth noting that $\alpha ^*=0.6$ (calculated by exhaustive search) is close to the spring coefficient, which was experimentally reached by try-and-error^1^. Interestingly, similar to the research by Collins *et al.*^5^, the metabolic cost versus the spring coefficient in Fig. 2 is a quadratic function.

### Biological joints’ moments

**Figure 3:**
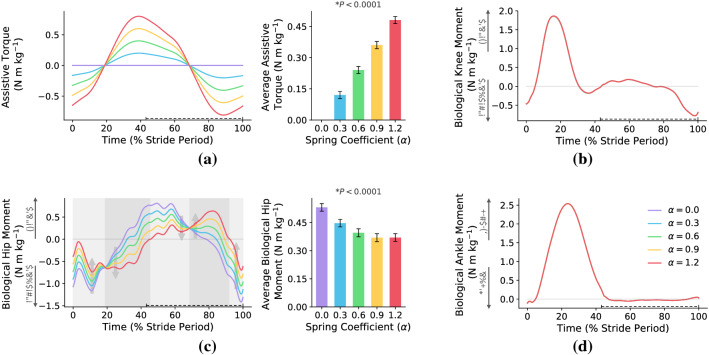
**Joints’ torques**. This figure shows the effects of exoskeleton augmentation on the joints’ biological moments and their average over the whole stride. The start of the gait cycle is at the heel-strike instant of the right leg, and all of the figures are for the right leg. The swing phase is shown with a dashed line over the horizontal axes. (**a**) is the exoskeleton torque profile for different stiffness coefficients. (**b**–**d**) show the biological knee, hip, and ankle moments. As it is clear, the exoskeleton augmentation only affects the biological hip moment, and the moments of the knee and ankle are not changed; thus, we have not plotted the average torque of the knee and ankle joints. The background color code for (**c**) is: light and dark gray mean biological hip moment improvement and disruption/increment, respectively. Moreover, the minimum of the average biological hip moment is on $\alpha =1$ where it has $31\%$ reduction compared to no exoskeleton case. And, the reduction of the average biological hip moment for $\alpha ^*=0.6$ is $26\%$. N $=7$; bars, mean; error bars, s.e.m; *P* values, ANOVA (random effect: participant; fixed effects: spring coefficient; significance level $=0.05$).

Here, we intend to analyze the relationship between metabolic rate reduction and average biological moment reduction. Accordingly, we plot the biological moment of all joints (hip, knee, and ankle) versus the gait cycle at different spring coefficients in Fig. 3. As can be seen, only the hip torque profile is affected by exoskeleton augmentation, and the torque profiles of the other joints (knee and ankle) remain almost fixed. This is an obvious observation since the kinematic of the gait is assumed to be a fixed function of time with/without exoskeleton torque augmentation at the hip joint; see the joint kinematics in Fig. 9.

Based on Fig. 3, compared to the no exoskeleton case ($\alpha =0$), the exoskeleton augmenting ($\alpha >0$) disturbs or improves the biological hip moment in the dark and light gray regions, respectively. In the dark gray regions, exoskeleton torque augmentation either increases the amplitude of biological hip moment or reverses its sign. Increasing the hip biological moment increases the muscles’ fatigue. And, any variation in the sign of the biological moment changes the muscles’ activation patterns; the muscle activation pattern is the timing that muscles are activated or remain silent. In light gray regions, the exoskeleton torque augmentation ($\alpha >0$) reduces the biological hip moment while its sign remains the same, which is the best possible condition for improving the muscles’ fatigue without any changes in the muscles’ activation patterns. Although dark gray and light gray regions are the same for all $\alpha >0$, for a certain spring coefficient ($\alpha =1$) we have on average the most biological hip moment reduction; $\alpha =1$ is the solution for inverse dynamics torque minimization.

Based on this observation, the spring coefficient ($\alpha ^*=0.6$) that minimizes the metabolic rate is much lower than the one which minimizes the biological hip moment ($\alpha =1$). In fact, this simulation supports the hypothesis that ”a moderate stiffness is sufficient for metabolic rate reduction”^5^. In other words, the stiffness coefficient that minimizes the metabolic rate is far lower than the value that minimizes the average biological hip moment. The reason for this difference relies on the overall scope of the average biological hip moment index. Reducing the average biological moment does not care about the biomechanics of the body. It is due to the existence of bi-articular muscles, antagonistic behavior, and high redundancy in the number of muscles. Therefore, in the next subsection, we check if minimizing individual muscles’ forces results in a better estimation of the optimum spring coefficient of *I-RUN*.

### Individual muscles’ forces

**Figure 4:**
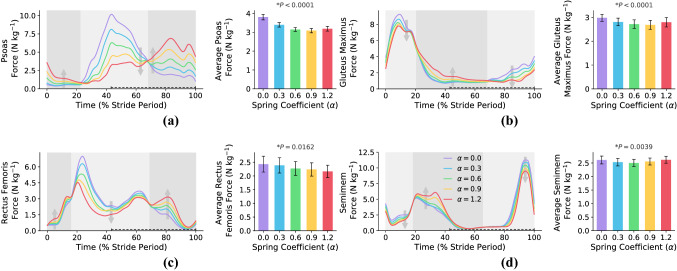
**Hip muscles’ forces**. This figure shows the effects of exoskeleton augmentation on the main hip muscles’ forces and their average over the whole stride. The start of the gait cycle is at the heel-strike instant of the right leg, and all of the figures are for the right leg. The swing phase is shown with a dashed line over the horizontal axes. The background color code for all of the sub-figures is: light(dark) gray means muscle force reduction(increment). As can be seen, exoskeleton augmentation changes the force profiles of all hip muscles. (**a**, **b**) show main hip antagonistic mono-articular muscles where Psoas is a hip flexor, and Gluteus Maximus is a hip extensor. The minimum of average Psoas and Gluteus Maximus forces are on $\alpha =0.8$, which yields to $19\%$ and $10\%$ reductions. And, the reductions of average Psoas and Gluteus Maximus forces for $\alpha ^*=0.6$ are $17.4\%$ and $8.9\%$, respectively. (**c**, **d**) Show main hip antagonistic bi-articular muscles where Rectus Femoris and Semimembranosus are hip flexor and extensor, respectively. The minimum of average Rectus Femoris and Semimembranosus forces are on $\alpha =1.4$ and $\alpha =0.6$, which yields to $11.7\%$ and $4.6\%$ reductions. And, the reductions of average Rectus Femoris and Semimembranosus forces for $\alpha ^*=0.6$ are $6.5\%$ and $4.2\%$, respectively. N $=7$; bars, mean; error bars, s.e.m; *P* values, ANOVA (random effect: participant; fixed effects: spring coefficient; significance level $=0.05$).

**Figure 5:**
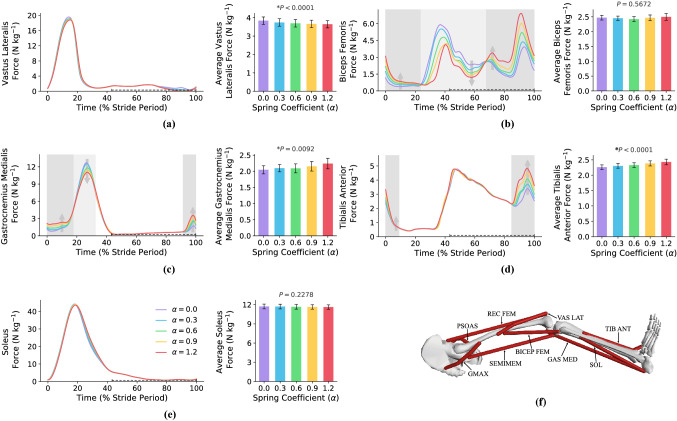
**Other muscles’ forces**. (**a**–**e**) Sub-figures show the effects of exoskeleton augmentation on the main muscles’ forces and their average over the whole stride except for the main hip muscles. The start of the gait cycle is at the heel-strike instant of the right leg, and all of the figures are for the right leg. The swing phase is shown with a dashed line over the horizontal axes. The background color code for all of the sub-figures is: white means no special effect, light gray means muscle force reduction, and dark gray means muscle force increment. As can be seen, the force of Soleus and Vastus Laterialis have no significant changes by exoskeleton augmentation. Besides, despite the significant changes in the Bicep Femoris force profile, the average force of this muscle shows no significant change in different spring coefficients; minimum on $\alpha =0.6$ with $2\%$ reduction. In addition, the optimum spring coefficient for Gastrocnemius Medialis and Tibialis Anterior is on $\alpha =0$. And, the minimum average force for Vastus Lateralis and Soleus is on $\alpha =1.5$ with $5.6\%$ and $1.2\%$ reductions compared to no exoskeleton case. Moreover, the average forces of these muscles have $3.8\%$ and $0.5\%$ reductions on $\alpha ^*=0.6$, respectively. N $=7$; bars, mean; error bars, s.e.m; *P* values, ANOVA (random effect: participant; fixed effects: spring coefficient; significance level $=0.05$). (**f**) shows the placement of the nine main branches of the muscles in the lower limb.

In this subsection, we study if there is a correlation between the metabolic rate reduction and individual muscles’ forces trajectory and their average over one gait cycle. To do so, the force profiles of nine main muscle branches in the presence of exoskeleton augmentation are illustrated in Figs. 4 and Fig. 5. Figure 4 shows the four main muscles contributing at the hip joint (Psoas in Fig. 4a, Gluteus Maximus in Fig. 4b, Rectus Femoris in Fig. 4c, and Semimembranosus in Fig. 4c) and the other five muscles are plotted in Fig. 5 (Vastus Laterialis in Fig. 5a, Bicep Femoris in Fig. 5b, Gastrocnemius Medialis in Fig. 5c, Tibialis Anterior in Fig. 5d, and Soleus in Fig. 5e). The exact placement of the nine main muscle branches are shown in Fig. 5f.

For hip muscles (Fig. 4), the exoskeleton augmentation leads to flexor muscles’ (Psoas and Rectus Femoris) forces increment approximately between $0\%-20\%$ and $70\%-100\%$ of the gait cycle while the extensor muscles’ (Gluteus Maximus and Semimembranosus) forces are reduced in these phases. The reverse behavior can be seen for $20\%-70\%$ of the gait cycle where the hip flexor(extensor) muscles’ forces are reduced(increased). This behavior can be explained by comparing the exoskeleton torque profile in Fig. 3a with the hip muscles’ forces in Fig. 4; there is an interesting correlation between these two figures. Accordingly, exoskeleton flexion(extension) torque decreases the flexor(extensor) muscles’ forces while amplifies the extensor(flexor) pair antagonistic muscle at the same time. Analyzing the average force over one gait cycle w.r.t. the metabolic rate diagram does not provide a logical correlation for the metabolic rate reduction.

Figure 5 also shows the force of other muscles; those muscles which are not connected to the hip joint. Based on Fig. 5, except Bicep Femoris muscle, exoskeleton augmentation leads to insignificant changes in the force of the other muscles such that the forces of Vastus Lateralis and Soleus are almost fixed. Although exoskeleton augmentation does not affect the biological moment profile of knee and ankle joints (see Fig. 3), it minimally changes the muscles’ forces that are not connected to the hip joint. This is due to the function of bi-articular muscles in the lower limb joints; torque distribution over the whole muscles. Accordingly, any variation in a single joint biological moment changes the force profiles of whole lower limb muscles; the changes are reduced by getting away from the augmented joint.

Since the metabolic rate reduction has some relations with individual muscles’ average forces reduction (e.g., Semimembranosus), a force-based index may potentially provide us with a measure for the metabolic rate reduction. In the next subsection, we study the relation between the whole muscles’ fatigue (which is defined upon the muscles’ forces) and metabolic rate reduction.

### Whole muscles’ fatigue

The whole muscles’ fatigue is a unified index for muscles’ forces redundancy resolution, and the static optimization methods in OpenSim utilize this index^38^. Besides, momentum minimization does not consider force re-distribution among the muscles, while muscle fatigue minimization is a candidate index for redundancy resolution and force distribution among the muscles. Therefore, we check if the whole muscles’ fatigue index minimization leads to the maximum metabolic rate reduction. However, the problem of computing the whole muscles’ fatigue in physical experiments is that it requires the force profiles of all muscles. To resolve this issue, previously, we presented the hypothesis that ”reducing the fatigue of two antagonistic muscles is sufficient for whole muscles’ fatigue reduction”^31^. Accordingly, instead of using the forces of 92 muscles in the lower limb model, we can rely on the fatigue of two antagonistic mono-articular muscles at the targeted joint. However, this hypothesis was studied on a 2-DOF model with 6 muscles (four mono-articular and two bi-articular). Therefore, first, we should check if our hypothesis is valid in a complex and general model of the human lower limb with 29-DOF and 92 muscles.

**Figure 6:**
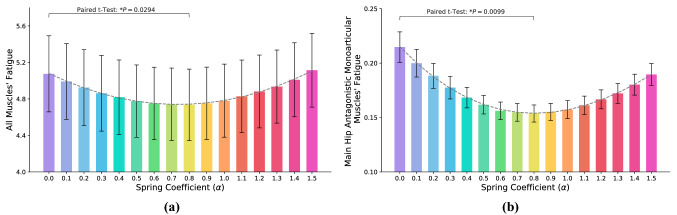
**Muscles’ fatigue**. (**a**) Shows all 92 muscles’ fatigue versus the stiffness coefficient (N $=7$; ANOVA; random effect: participant; fixed effects: spring coefficient and square of spring coefficient; $P_{scoef}=0.0343$, $P_{scoef^2}<0.0001$). The optimum spring coefficient for the whole muscles’ fatigue reduction is at $\alpha ^{\#}=0.8$ (paired two-sided *t* test with correction for multiple comparisons; $P=0.0294$). The dashed line is the quadratic best-fitted profile to average muscles’ fatigue ($R^2=0.99$, $P<0.0001$). (**b**) Shows main hip antagonistic mono-articular muscles fatigue versus the stiffness coefficient (N $=7$; ANOVA; random effect: participant; fixed effects: spring coefficient and square of spring coefficient; $P_{scoef}<0.0001$, $P_{scoef^2}<0.0001$). The optimum spring coefficient for the mono-articular muscles’ fatigue reduction is at $\alpha ^{\#}=0.8$ (paired two-sided *t* test with correction for multiple comparisons; $P=0.0099$). The dashed line is the quadratic best-fitted profile to average muscles’ fatigue ($R^2=0.99$, $P<0.0001$). Comparing (**a**) and (**b**) shows that stiffness coefficient optimization based on feedback from mono-articular muscles leads to whole muscles’ fatigue reduction.

**Figure 7:**
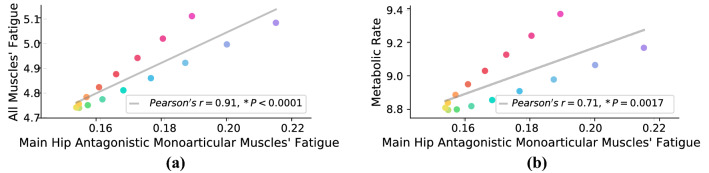
(**a**) is the correlation between all muscles’ fatigue and two antagonistic mono-articular hip muscles’ fatigue, and (**b**) is the correlation between metabolic rate and two antagonistic mono-articular hip muscles’ fatigue; the color of each point in this plot is the same with its column color in Fig. 6. The *P* value for (**a**, **b**) are $P<0.0001$ and $P=0.0017$, and ”Pearson’s r-value” are $r=0.91$ and 0.71. Both figures show a certain linear correlation between the parameters. Interestingly, for both figures, near to the optimum spring coefficient, the deviations from the linear estimation are minimum, and by getting away from the optimum stiffness coefficient, the deviations increase.

The whole muscles’ fatigue versus spring coefficient is plotted in Fig. 6a. In the hip joint, the main antagonistic mono-articular muscles are Psoas and Gluteus Maximus, and the fatigue of these muscles is also reported in Fig. 6b. Comparing Fig. 6a,b indicates that the average of ”all muscles’ fatigue” and ”two main hip antagonistic mono-articular muscles’ fatigue” are correlated functions of spring coefficient with the same optimum; the average mismatching error for 16 spring coefficient and all subjects is $0.89\pm 1.96 \%$ and the linear correlation criteria is $91\%$; see Figs. 6 and 7a. These results provide support for the correctness of our proposed hypothesis in a complex and generic model of the human lower limb. Using this hypothesis, one can minimize the whole muscles’ fatigue by utilizing the force of only two mono-articular antagonistic muscles. Accordingly, due to the monotonic relation between muscles’ forces and root-mean-square (RMS) of the EMG signals^46,47^, we can drastically reduce the number of EMG sensors required in practice; only two EMG sensors are sufficient for *I-RUN* stiffness adaptation. In addition, comparing the metabolic rate with the fatigue of two antagonistic mono-articular muscles (Fig. 7b) also indicates a linear relationship between these two parameters; i.e., the fatigue of two antagonistic mono-articular muscles can be used as an indicator for metabolic rate reduction as well.

Comparing Figs. 2 and 6 shows that the optimum stiffness coefficient suggested by muscles’ fatigue reduction is different from the value that minimizes the metabolic rate. However, compared to the other candidates, such as average biological hip moment and individual muscles’ fatigue, it suggests a much closer and reasonable solution for metabolic rate minimization. The suggested spring coefficient ($\alpha ^{\#}=0.8$) is a sufficient/reliable sub-optimal for metabolic rate reduction using only two EMG sensors. Compared to indirect calorimetry, EMG sensors are much easier to use. In addition, they provide us with faster dynamics that not only reduce the experiment time but also significantly improve the speed of adaptation methods compared to gas-analyzer-based approaches; e.g., human-in-the-loop^48,49^.

### A kinematic index for metabolic rate reduction

**Figure 8:**
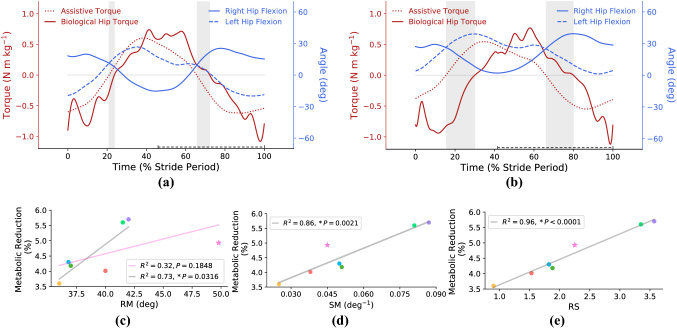
**Kinematic analysis**. (**a**, **b**) compare the normalized biological hip torques, hip joints’ angles, and normalized assistive torques for the subjects with the highest and lowest metabolic rate reductions. The start of the gait cycle is at the heel-strike instant of the right leg, and the torque profiles in (**a**) and (**b**) are for the right leg. The swing phase is shown with a dashed line over the horizontal axes. Gray areas define the difference between the zero points of the biological hip torque and assistive torque. As it is clear, gray areas are much narrower for the subject with the highest metabolic rate reduction. (**c**–**e**) Compare three different defined kinematic indexes for estimating the metabolic rate reduction. In figures (**c**–**e**), each color indicates one subject. As can be seen, *P* value and goodness of fit are improved from RM, SM, and RS $=$ RM $\times$ SM, respectively. In (**c**), the linear regression (pink line) cannot properly estimate the metabolic rate such that there is an outlier (pink star) in this figure; by excluding the outliers based on the Jack-knife criterion (significance level $=0.05$), the goodness of fit of the linear regression (gray line) is improved. However, in (**d**, **e**), the outlier approaches to the linear regression and the goodness of fit improves further. Accordingly, the best index for having a good linear relation between metabolic rate reduction and kinematic parameters is RS.

*I-RUN* torque is a linear function of the difference between hip angles; i.e., the gait kinematics directly changes the torque profile. Hence, the hip angle and gait kinematic have an undeniable effect on metabolic rate reduction. Accordingly, a kinematic index has more chance to show the harmony between the gait and the passive exoskeleton torque to maximize the metabolic rate reduction. As it is discussed in “Biological joints’ moments” section, a proper torque profile is the one that reduces the amplitude of the biological hip moment without changing its sign, which is different from the average biological hip moment reduction. To study the importance of gait kinematics on the exoskeleton torque profile, we compare the biological hip moment, exoskeleton torque, and hip angles of two extreme cases in Fig. 8a and Fig. 8b; i.e., the subjects with the highest and lowest metabolic rate reductions.

By comparing Fig. 8a,b in the gray regions, it is concluded that if the zero points of exoskeleton torque and the biological hip moment are close to each other, the exoskeleton torque reduces the amplitude of biological hip moment without changing its sign. The zero points of the exoskeleton torque are equivalent to the zero value of the difference of hip angles. Now the question is how we can change the zero point of exoskeleton torque by kinematic modification?

**Figure 9:**
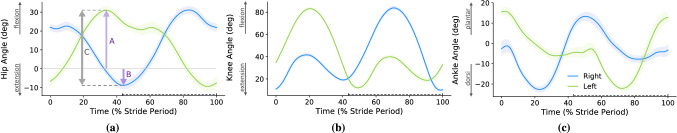
**Joints’ trajectories and definition of kinematic indexes**. In this figure, (**a**–**c**) show the hip, knee, and ankle joints’ trajectories, respectively. The start of the gait cycle is at the heel-strike instant of the right leg, and the swing phase is shown with a dashed line over the horizontal axes. Figure (**a**) shows the hip joint trajectory and the definition of kinematic indexes. In figure (**a**), *A* is the maximum angle of the left hip joint, *B* is the minimum angle of the right hip joint, and *C* is the absolute value of the difference between *A* and *B*. It is important to note that *A* and *B* are vectors and can be either positive or negative, and *C* is a distance, and it is always positive. As discussed “A kinematic index for metabolic rate reduction” section, the kinematic indexes are defined as range of motion (RM) = $C=|A-B|$, symmetry of motion (SM) = ${|A+B|}^{-1}$, and RS $=$ RM $\times$ SM = $C\times {|A+B|}^{-1}$; |*x*| means the absolute value of *x*.

There are two parameters that contribute to moving the zero point of exoskeleton torque towards the biological hip moment; (1) symmetry of motion (SM) and (2) range of motion (RM) which are depicted in Fig. 9a. The mathematical definitions of SM and RM are SM $=|\max \{q^l_h\}+\min \{q^r_h\}|^{-1}$ and RM $=|\max \{q^{l}_h\}-\min \{q^r_h\}|$ where $q^l_h$ and $q^r_h$ are the angles of the left and the right hip joints. We also define another index (RS) which considers both symmetric motion (SM) and range of motion (RM) at the same time as RS $=$ RM $\times$ SM.

To study the relation between RM, SM, RS, and metabolic rate reduction, we plot the metabolic rate reduction of each individual subject (at the optimum stiffness coefficient $\alpha ^*=0.6$) versus RM, SM, and RS in Fig. 8c, d, and Fig. 8e, respectively. In these figures, the metabolic rate reduction is estimated using linear regressions. In Fig. 8c, the starred subject (shown by the pink star) is an outlier for the linear estimation. Accordingly, a second linear regression line is also plotted by excluding the outlier subject. However, interestingly, the starred subject is not an outlier for the SM and RS indexes. Hence, compared to RM, SM and RS are better indexes for estimating metabolic rate reduction. And, among all of the indexes, RS provides the best linear estimation for all of the subjects, including the starred one. Therefore, it is concluded that RS is a rich and representative index for estimating the metabolic rate reduction. It is noteworthy to consider that RS is computed only for one stride. Obviously, if the subject performs a rhythmic running gait, these indexes are the same for whole strides. And, in the case that the subject performs a non-rhythmic running gait, the indexes of each stride should be studied individually; the overall indexes are the average indexes in each stride.

RS index provides a systematic monitoring approach to potentially teach the users how to maximize their metabolic rate reduction by improving gait kinematic (RS index). However, there is a concern that changing the gait kinematic may change the optimum spring coefficient in the long-term. By checking the minimum metabolic rate for each individual, it is concluded that regardless of the subject’s kinematic, the optimum spring coefficient for most of the subjects is $\alpha ^*=0.6$. Hence, the optimum spring coefficient is independent of the subject’s kinematic, but the amount of metabolic rate reduction is a linear function of the RS index. In other words, the RS index (gait kinematic) can be used as a training metric to maximize individuals’ metabolic rate reduction, while changing their gait kinematic does not change the optimum spring coefficient.

**Figure 10:**
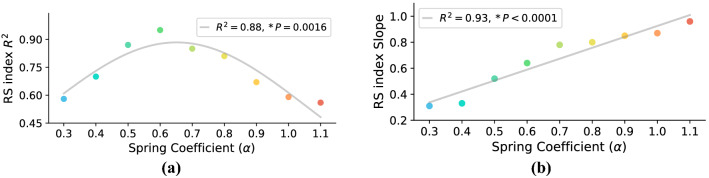
To check the linear relation between RS index and metabolic rate reduction in all spring coefficients, in this figure, we plot the $R^2$ (goodness of fit measure) and slope of this linear regression (gray line in Fig. 8e) for different spring coefficients. (**a**) Shows that the $R^2$ of linear regression, which is a quadratic diagram, and figure (**b**) indicates a linear relationship between the slope of linear regression and spring coefficients. It is worth mentioning that for the spring coefficients $\alpha <0.3$ and $\alpha >1.1$, $R^2$ is very low; there is no linear relation for these ranges of $\alpha$. (**b**) indicates that by increasing the spring coefficient, the variations of metabolic rate w.r.t. RS index increases; i.e., the sensitivity of the metabolic rate versus RS index increases.

Another interesting study is to check if the RS index can also be used to predict the metabolic rate reduction of individuals with the spring coefficients rather than the optimum one. To study this point, we have tried to estimate the metabolic rate reduction for each spring coefficient as a function of the RS index. The $R^2$ (goodness of fit measure) and slope of this linear regression v.s. different spring coefficients are presented in Fig. 10a, b, receptively. Figure 10a shows that metabolic rate reduction is a linear function of RS index for spring coefficients between $\alpha =0.3$ and $\alpha =1.1$. Interestingly, the highest $R^2$ is at $\alpha ^*=0.6$, which means for the optimum spring coefficient, the metabolic rate is almost a linear function of the RS index. Figure 10b also shows that in all of the spring coefficients, the slope of the linear relation is positive; i.e., increasing RS index increases the metabolic rate reduction. In addition, it is concluded that by increasing the spring coefficient, variation of metabolic rate w.r.t. the RS index increases; the sensitivity increases.

RS is just a kinematic index and can be measured easily compared to metabolic rate and EMG signals. It provides a metric not only for estimation of biological hip moment compensation and metabolic rate reduction but also for analyzing the quality of the subject’s training sessions. Using the proposed index, one can supervise the process of the training with the exoskeleton and lead the subjects to maximize their benefit from *I-RUN*. This index can also explain the individual differences in the metabolic rate reduction of the experiments as well as an improvement over the sessions. Nevertheless, these findings are based on some simulations, and extensive experiments are required for physical validations, which is considered our future work.

## Conclusion

In this paper, we presented a simulation-based biomechanical assessment of passive assistive torque augmentation at the hip joint. The case study for this simulation assessment was our previously designed exoskeleton, named *I-RUN*. The simulation study was done at the running speed of 2*m*/*s* on a generic model of a human lower limb with 92 Hill-type muscles and 29-DOF in OpenSim software. At the first step, we computed the optimal spring coefficient for *I-RUN* exoskeleton. The optimal spring coefficient is close to one, which we previously reported in the *I-RUN* paper. At the next step, we showed that the best assistive torque profile for reducing the joint biological moment and muscles’ force is the assistive torque profile that minimizes the biological torque without changing its sign. Furthermore, we observed that the gradient of fatigue of only two mono-articular muscles at the hip joint is in the same direction as the gradient of all 92 muscles’ fatigue for the lower limb. This interesting observation proves the generality of the previously proposed hypothesis that ”reducing the forces of two antagonistic mono-articular muscles is sufficient for total muscles’ fatigue reduction.” At the final step of our biomechanical assessment, it is observed that the kinematic of the hip joint does not have a significant impact on the optimal spring coefficient; however, it significantly affects the amount of metabolic rate reduction. Based on these observations, a kinematic index is presented to predict the metabolic rate reduction by the *I-RUN* augmentation. This metric can be used for training individuals to maximize their metabolic reduction while using the *I-RUN*; however, this metric needs to be validated in experiments, which is considered as our future work.

## Data Availability

The data used in this study were originally generated by Hamner and Delp (2013), which is freely available at https://simtk.org/home/nmbl_running. All data generated or analyzed during this study are included in the article.
